# The Mouse Cohesin-Associated Protein PDS5B Is Expressed in Testicular Cells and Is Associated with the Meiotic Chromosome Axes

**DOI:** 10.3390/genes1030484

**Published:** 2010-12-13

**Authors:** Tomoyuki Fukuda, Christer Hoog

**Affiliations:** Department of Cell and Molecular Biology, Karolinska Institute/Berzelius vag 35, 171-77 Stockholm, Sweden; E-Mail: christer.hoog@ki.se

**Keywords:** meiosis, chromosome, cohesin, PDS5, synaptonemal complex

## Abstract

During the first meiotic prophase, the cohesin complex is localized to the chromosome axis and contributes to chromosome organization, pairing, synapsis, and recombination. The PDS5 protein, an accessory factor of the cohesin complex, is known to be a component of meiotic chromosome cores in fungi and to be implicated in meiotic chromosome structure and function. We found by immunoblotting experiments that a mammalian PDS5 protein, PDS5B, is abundantly expressed in mouse testis compared to other tissues. Immunofluorescence labeling experiments revealed that PDS5B is highly expressed in spermatogonia and that most PDS5B is depleted from chromatin as cells enter meiosis. During the first meiotic prophase, PDS5B associates with the axial cores of chromosomes. The axial association of PDS5B was observed also in the absence of synaptonemal complex proteins, such as SYCP1 and SYCP3, suggesting that PDS5B is an integral part of the chromosome axis as defined by the cohesin complex. These results suggest that PDS5B modulates cohesin functions in spermatocytes as well as in spermatogonia, contributing to meiotic chromosome structure and function.

## 1. Introduction

Meiosis is the process by which diploid cells produce haploid gametes, sperm and eggs, or spores capable of sexual reproduction. During meiosis, two rounds of chromosome segregations occur after only a single round of DNA replication to reduce the number of chromosomes in half. At the first meiotic division, homologous chromosomes (homologs) separate from each other, while sister chromatids separate at the second meiotic division. For the first meiotic division to occur properly, homologs must be aligned, synapsed, and then linked through crossover recombination during the first meiotic prophase [1,2].

At the beginning of the first meiotic prophase, recombination is initiated by SPO11 protein-mediated double-strand breaks (DSBs) in genomic DNA [3]. The formation of DSBs is required for the initial alignment of homologs, a process called pairing, in some organisms including the mouse [4]. After pairing, a proteinaceous structure called the synaptonemal complex (SC) is established along the homologs [5,6]. SC formation is initiated by the establishment of axial elements (AEs) along the chromosome axis. Each homolog pair is then joined by transverse filaments (TFs) along the entire length of the AEs (referred to as LEs, lateral elements, in the context of the SC) to produce the mature SC. Formation of the SC is required for the completion of recombination to produce crossover products [7]. After disassembly of the SC, homologs are physically linked only at the sites of crossover, called chiasmata, which ensure the proper orientation and faithful segregation of homologs at the first meiotic division [1,2].

The cohesin complex holds sister chromatids together until their segregation at mitosis or meiosis [1,2]. The mitotic cohesin complex consists of SMC1α, SMC3, stromal antigen protein 1/2 (STAG1/2 or SA1/2), and RAD21 in mammals [8]. The meiotic cohesin complex additionally contains several meiosis-specific components, including SMC1β, STAG3, and REC8 [8]. During the first meiotic division, cohesins are removed from chromosome arms in order to promote the segregation of homologs [1,2]. Cohesin at centromeres is protected at this time point, allowing sister chromatids to be connected until the second meiotic division [1,2]. The centromeric cohesin is subsequently removed, resulting in the separation of sister chromatids at the second division.

Besides the role in sister chromatid cohesion, the cohesin complex also plays critical roles in meiotic chromosome structure and function. During the first meiotic prophase, the cohesin complex localizes along the entire axial cores of chromosomes [8]. In mammals, cohesins are associated with the residual chromosome axes even in the absence of AE proteins, SYCP2 and SYCP3 [9]. Moreover, deletion of genes coding for meiosis-specific cohesin subunits changes chromosome axis organization [10,11,12,13]. Thus, the cohesin complex has an important structural role during the first meiotic prophase. In addition, the absence of meiotic cohesin components also causes defects in pairing, SC formation, and recombination in many organisms including the mouse, suggesting that the cohesin complex has multiple functions during meiotic prophase [10,11,12,14]. 

PDS5 is a cohesin-associated protein that regulates maintenance as well as removal of the mitotic cohesin complex [15,16,17,18,19]. Like the cohesin complex, PDS5 is known to be associated with meiotic chromosomes during the first prophase in fungi [20,21,22,23]. In fungi and worms, lack of PDS5 causes defects in chromosome morphology, pairing, synapsis, recombination, and cohesion during meiosis [20,21,22,23,24]. Vertebrates have two PDS5 proteins, PDS5A and PDS5B [18]. Mice lacking PDS5A or PDS5B have no cohesion defects but exhibit developmental defects that are thought to reflect the role of the cohesin complex in regulating gene expression [25,26]. Although PDS5A is dispensable for gametogenesis in mice [26], *Pds5B*-deficient mice have severe reduction in primordial germ cells (PGCs) in testes and ovaries [25]. This defect prohibits a genetic analysis of the role of PDS5B at later stages of gametogenesis. To gain more insights into the roles of the PDS5 proteins in mammalian germ cells, we analyzed the temporal and spatial expression of PDS5B in mouse testis. We found that the PDS5B protein is associated with the meiotic chromosome axis during the first meiotic prophase, suggesting a role for the mammalian PDS5 in meiotic chromosome structure and function.

## 2. Results and Discussion

To investigate the expression of the PDS5B protein in mouse testicular cells, we performed immunoblotting and immunofluorescence labeling analyses.

### 2.1. PDS5B Is Highly Expressed in Mouse Testis

**Figure 1:**
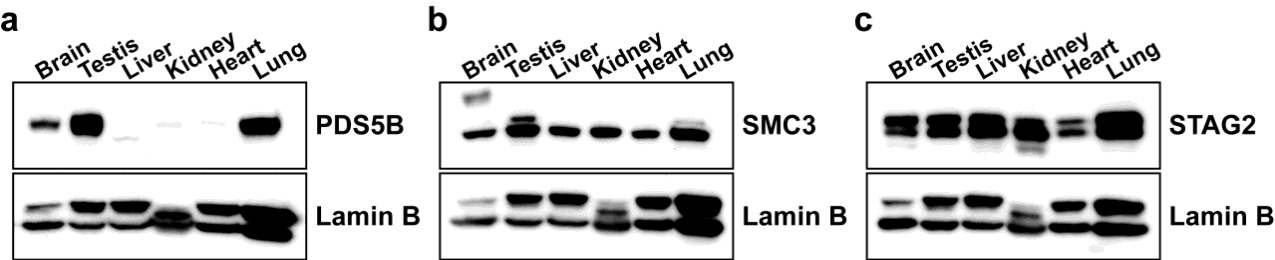
Expression of PDS5B and cohesin components in various mouse tissues. Approximately 35 μg of nuclear extract proteins prepared from tissues were loaded and probed with antibodies against PDS5B (**a**), SMC3 (**b**), and STAG2 (**c**). Lamin B was used as a nuclear loading control.

The expression of PDS5B was surveyed in adult mouse tissues by immunoblotting. Among tissues examined, protein expression levels of PDS5B were found to be highest in testis and lung (Figure 1a). PDS5B was also abundant in brain (Figure 1a). Our data are consistent with the reported highest expression of *Pds5B* mRNA in testis and brain [25]. Interestingly, the expression pattern of PDS5B contrasts with that of the cohesin components, SMC3 (Figure 1b) and STAG2 (Figure 1c), which are much more uniformly expressed in the analyzed tissues, suggesting that PDS5B modulates cohesin function in a tissue-specific manner.

### 2.2. PDS5B Is Associated with the Meiotic Chromosome Axis

**Figure 2:**
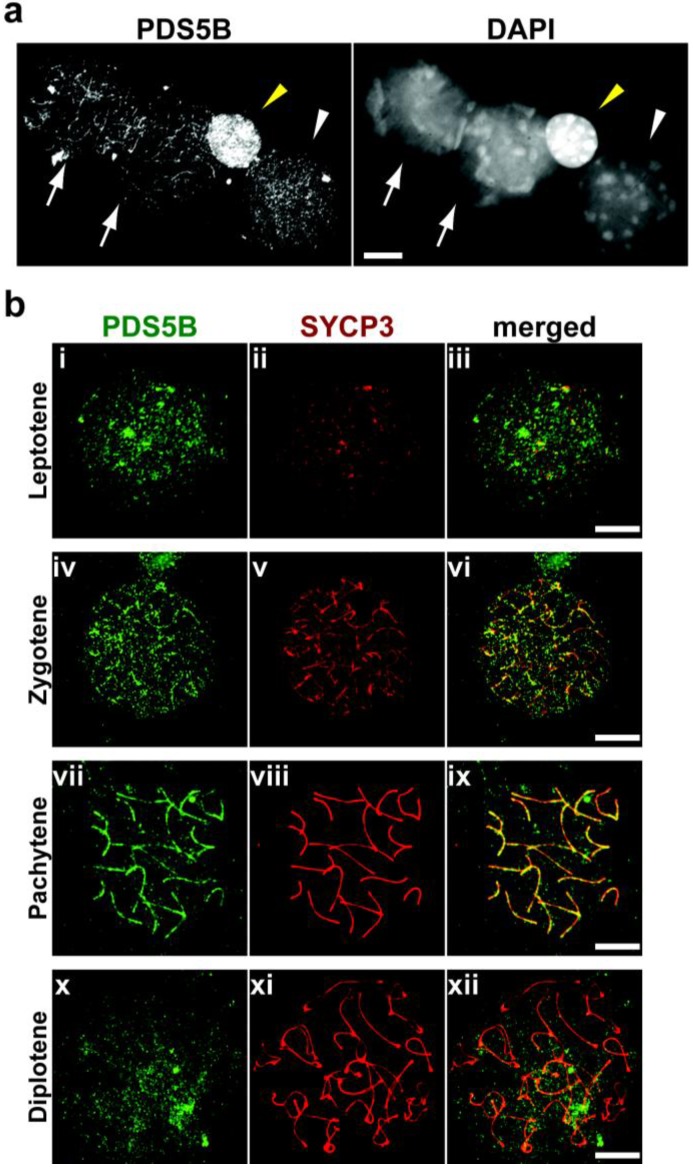
Localization of PDS5B in testicular cells. (**a**) Nuclear spreads of testicular cells stained with anti-PDS5B antibody (left) and DAPI to visualize DNA in nuclei (right). Arrows, pachytene spermatocytes; yellow arrowhead, spermatogonium; white arrowhead, preleptotene spermatocyte. Bar, 10 μm; (**b**) Nuclear spreads of leptotene (I to iii), zygotene (iv to vi), pachytene (vii to ix), and diplotene (x to xii) spermatocytes stained with antibodies against PDS5B (green) and an AE component, SYCP3 (red). Bars, 10 μm.

To determine whether PDS5B is associated with the chromosome in testicular cells, we immunostained surface-spread chromosomes for PDS5B and an AE component, SYCP3. The PDS5B signal was found to be strong on chromatin in spermatogonia (Figure 2a), but less strong in spermatocytes (Figure 2a), indicating that the presence of PDS5B on chromatin is reduced as cells enter meiosis. When the AEs start to assemble at the leptotene stage, PDS5B is first observed on chromatin (Figure 2b, i to iii), similar to what is seen at the preleptotene stage (Figure 2a). At zygotene, when the AE is fully developed and the homologs have initiated synapsis, the PDS5B signal starts to be detected on the chromosome axis as marked by SYCP3 (Figure 2b, iv to vi). The axial association of the PDS5B signal culminates at the pachytene stage when the homologs are completely synapsed (Figure 2b, vii to ix). Subsequently, the PDS5B signal is depleted from the chromosome axis when homologs undergo desynapsis at diplotene (Figure 2b, x to xii). We could not observe PDS5B on the chromosomes after diplotene (data not shown). The expression of PDS5B in spermatogonia and spermatocytes is consistent with the reported expression pattern of *Pds5B* mRNA in adult testis: *Pds5B* is highly expressed at the periphery of the seminiferous tubules where spermatogonia and spermatocytes are located [25].

**Figure 3:**
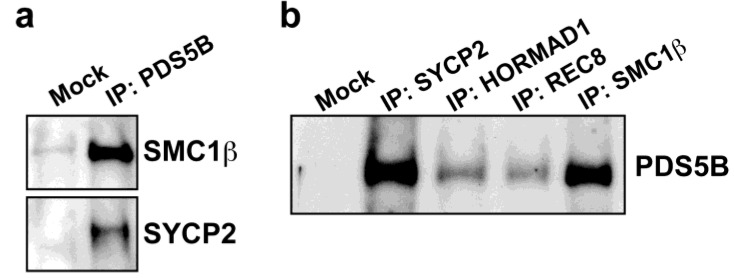
PDS5B is associated with the meiotic chromosome axis. (**a**) Proteins prepared from testis were immunoprecipitated with protein A beads only (Mock) or with protein A beads coupled to anti-PDS5B antibody (IP). Immunoprecipitates were probed with antibodies against a cohesin component, SMC1β and an AE component, SYCP2; (**b**) Proteins prepared from testis were immunoprecipitated with protein A beads (Mock) or with protein A beads coupled to antibodies against chromosome axis components. Immunoprecipitates were probed with anti-PDS5B antibody.

The axial association of PDS5B was confirmed by immunoprecipitation assays. Testis protein extracts were immunoprecipitated against PDS5B. In the immunoprecipitates, SMC1β, a meiosis-specific cohesin component, and SYCP2, an AE protein, were detected, supporting the notion that PDS5B is associated with the meiotic chromosome axis. Conversely, PDS5B was detected in immunoprecipitates of meiotic axis proteins, such as SYCP2 and HORMAD1, and meiosis-specific cohesin components, such as REC8 and SMC1β (Figure 3b). Thus, we conclude that PDS5B is associated with the meiotic chromosome axis and, either directly or indirectly, with cohesins.

### 2.3. Axis Association of PDS5B Is Independent of SC Proteins

**Figure 4:**
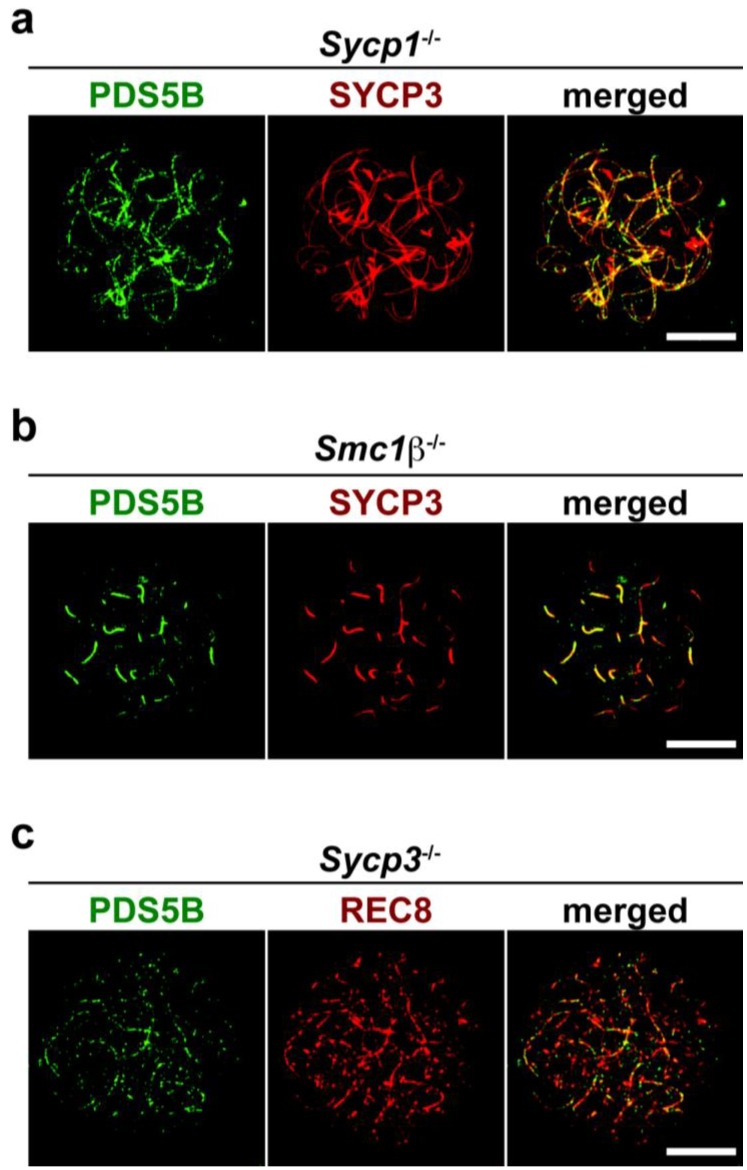
PDS5B localization in synaptonemal complex (SC) mutants. (**a**) *Sycp1*^-/-^ spermatocyte stained with antibodies against PDS5B (green) and SYCP3 (red); (**b**) *Smc1β*^-/-^ spermatocyte stained with antibodies against PDS5B (green) and SYCP3 (red); (**c**) *Sycp3*^-/-^ spermatocyte stained with antibodies against PDS5B (green) and a cohesin component, REC8 (red). Bars, 10 μm.

To investigate the relationship between PDS5B and other axial core proteins, the axis association of PDS5B was assessed in mutant mice lacking meiotic chromosomal proteins. First, we immunostained PDS5B in *Sycp1*^-/-^ spermatocytes to test whether synapsis of homologs is required for the chromosome association of PDS5B. SYCP1 is a TF protein, in the absence of which homologs are paired but not synapsed [27]. PDS5B was observed on the aligned but unsynapsed axes in the *Sycp1* mutant spermatocytes (Figure 4a). This result reveals that chromosome synapsis is not necessary for the chromosomal localization of PDS5B during the first meiotic prophase.

Next, we assessed the PDS5B localization in *Smc1β*^-/-^ spermatocytes. In the absence of SMC1β, the meiotic chromosome axis is formed by AEs and the remaining SMC1α-type cohesin complex, but chromosome axes are hyper-compacted and synapsis is partially impaired [11]. In nuclear spreads of the *Smc1β*^-/-^ mutant, PDS5B was found to be associated with short chromosome axes (Figure 4b). As in wild-type cells, the PDS5B signal is prominent at synapsed chromosomes that are marked by the relatively strong signal of SYCP3 (Figure 4b). Thus, in the absence of SMC1β, PDS5B is associated with chromosome axes formed by AE proteins and other cohesin components.

We also examined spermatocytes derived from an *Sycp3*^-/-^ mutant to define whether PDS5B is dependent on AE formation for its localization to the chromosome axis. In *Sycp3*^-/-^ meiocytes, AE structures consisting of SYCP3 and SYCP2 are absent, but the residual chromosome axis is formed by the cohesin complex [9]. We immunostained surface-spread chromosomes of *Sycp3*^-/-^ spermatocytes for PDS5B. A meiosis-specific cohesin component, REC8, was used to visualize the cohesin cores. As shown in Figure 4c, PDS5B is associated with the chromosome axis formed by the cohesin complex in the absence of AEs. This result is consistent with the fact that PDS5B is a cohesin-binding protein, suggesting that the mammalian PDS5 may associate with and regulate the cohesin complex on the meiotic chromosome axis.

### 2.4. Possible Roles of the PDS5B Protein in Spermatogenesis

In adult mouse testis, PDS5B is primarily expressed in spermatogonia (Figure 2a). It has been suggested that PDS5B is required for proliferation of PGCs [25]. Like in PGCs, PDS5B may be important for proliferation in spermatogonia. Alternatively, PDS5B may function in cell differentiation through its non-cohesion function.

PDS5B is associated with the meiotic chromosome axis (Figure 2 and Figure 3). This is consistent with meiotic localization of the PDS5 protein in other organisms [20,21,22,23]. In yeast, chromosomal association of PDS5 depends on the cohesin complex but that of the cohesin complex is independent of PDS5 [15,16,17,21,23]. We here reveal that PDS5B is associated with cohesin cores in the absence of AEs (Figure 4c). Therefore, we suggest that PDS5B localizes to the meiotic chromosome axis through its association with cohesin in a manner conserved among eukaryotes. As in fungi [22,23], PDS5B may be required for chromosome morphogenesis, paring, synapsis, and recombination via the regulation of cohesin.

PDS5 proteins have been proposed to contribute to the resolution of sister chromatid cohesion [19], as well as for the maintenance of cohesion [15,16,17,18]. In addition to PDS5, eukaryotes have another conserved cohesin-associated protein, WAPL [28,29]. PDS5 physically interacts with WAPL [28,29] and it has been proposed that they together modulate the cohesin complex to promote its dissociation from the chromatin [19,30,31]. Like PDS5B, mammalian WAPL is highly expressed in testis and is associated with the meiotic chromosome axis [32,33]. On the other hand, another conserved factor, shugoshin protects the cohesin complex from dissociation at kinetochores [34,35,36] and chromosome arms [19]. Thus, it is possible that PDS5, WAPL, and shugoshin co-regulate association and dissociation of the cohesin complex on the chromosome axis during the first meiotic prophase. These activities may facilitate the maintenance of cohesin core structures, dissociation of the SC at diplotene, or modulation of chromosome integrity at recombination sites. Conditional null alleles of *Pds5B* will allow us to address the function of PDS5B in testicular cells.

## 3. Experimental Section

### 3.1. Animals

C57BL/6 wild-type, *Sycp1*^-/-^ [27], *Smc1β*^-/-^ [11], and *Sycp3*^-/-^ [37] mice were used and maintained according to regulations provided by the animal ethical committee of the Karolinska Institutet, which also approved the experiments.

### 3.2. Immunoblotting

Nuclear extracts were prepared as described previously [38]. Briefly, each tissue was homogenized in a buffer containing 0.32 M Sucrose, 10 mM HEPES pH 7.4, 1 mM phenylmethylsulfonyl fluoride (PMSF), and the complete protease inhibitor cocktail (Roche). After centrifugation at 1,000 g, the pellet was resuspended in RIPA buffer (50 mM Tris-HCl, pH 7.5, 150 mM NaCl, 1 mM EDTA, 1% NP-40, 0.5% Na-deoxycholate, 0.1% SDS, and protease inhibitors). After sonication and centrifugation at 16,000 g, the supernatant was recovered as nuclear extracts. The protein concentration in the extracts was determined by the BCA protein assay (Pierce). Extracts or immunoprecpitates were separated by SDS-PAGE, transferred to a PVDF membrane (Millipore), and probed with antibodies against PDS5B (Cat#IHC-00381, Bethyl Laboratories, 1:1,000), SMC3 (Abcam, 1:1,000), STAG2 (Abcam, 1:1,000), Lamin B [39] (1:4,000), SMC1β [40] (1:1,000), and SYCP2 [40] (1:1,000). The specificity of the anti-PDS5B antibody was confirmed by immunoblotting and immunoprecipitation using another anti-PDS5B antibody (Cat#A300-538A, Bethyl Laboratories) (see Supplementary Figure S1).

### 3.3. Immunoprecipitation

For immunoprecipitation, testes were homogenized in a buffer containing 0.32 M Sucrose, 10 mM HEPES pH 7.4, 1 mM PMSF, and the complete protease inhibitor cocktail (Roche). After centrifugation at 1,000 g, the pellet was resuspended in IP buffer (25 mM Tris-HCl, pH 7.5, 150 mM NaCl, 5 mM EDTA, 1% Triton X-100, 0.5% Na-deoxycholate, 0.1% SDS, and protease inhibitors). After centrifugation at 16,000 g, the pellet was resuspended in IP buffer, sonicated, and centrifuged at 16,000 g. The supernatant was pre-cleared with protein A-conjugated Dynabeads (Invitrogen), and then incubated with antibodies against PDS5B (Bethyl Laboratories), SYCP2 [40], HORMAD1 [38], REC8 [40], or SMC1β [40] bound to protein A-conjugated Dynabeads (Invitrogen). Immunoprecipitates were washed in RIPA buffer, eluted in LDS protein sample buffer (Invitrogen), and subjected to immunoblotting.

### 3.4. Nuclear Spreads

Preparation of nuclear spreads was performed according to the methods for surface spreading of meiotic chromosomes described previously [41]. To detect proteins, we used the following antibodies and dilutions: rabbit anti-PDS5B (Cat#IHC-00381, Bethyl Laboratories), 1:50; mouse anti-SYCP3 (Santa Cruz Biotechnology), 1:400; guinea pig anti-REC8 [40], 1:100. Signals were visualized by donkey anti-rabbit Alexa Fluor 488 (Invitrogen, 1:1,000), donkey anti-mouse Cy3 (Jackson ImmunoResearch, 1:1,200) and donkey anti-guinea pig Cy3 (Jackson ImmunoResearch, 1:1,200). Slides were viewed at room temperature using a Leica DMRA2 microscope. Images were captured with a Hamamatsu digital charge-coupled device camera C4742-95 and Openlab 3.1.4 software (Improvision). Images were processed using Adobe Photoshop CS2. The specificity for the PDS5B staining was confirmed by competing with the peptide corresponding to the epitope of the anti-PDS5B antibody (Supplementary Figure S2).

## 4. Conclusions

PDS5 proteins are known to associate with the chromosomal axes during the first meiotic prophase in fungi. Mice lacking PDS5B exhibit severe reduction in PGCs, indicating a critical role of PDS5B during spermatogenesis [25]; however, the function of PDS5B during meiosis remains unclear. In this study, we reveal that the PDS5B protein is expressed in spermatogonia and spermatocytes and that PDS5B is associated with the axial cores of chromosomes at the first meiotic prophase (Figure 2 and Figure 3). The axial association is independent of SC or AE formation (Figure 4), supporting the notion that PDS5B is part of the cohesin cores. Thus, it is suggested that mammalian PDS5 has a conserved role in chromosome axis organization and function during meiosis.

## Supplementary Files

Supplementary File 1 PDF-Document (PDF, 132 KB)
