# Supplementary material for: The Mouse Cohesin-Associated Protein PDS5B Is Expressed in Testicular Cells and Is Associated with the Meiotic Chromosome Axes
Source: Genes (Basel). 2010 Dec 13;1(3):484–94. doi: 10.3390/genes1030484 (PMC3966231; doi:10.3390/genes1030484)

Supplementary Figure S1. Immunoprecipitation (IP) and immunoblotting (IB) of PDS5B using two different anti-PDS5B antibodies, #1 (Cat#IHC-00381, Bethyl Laboratories) and #2 (Cat#A300-538A, Bethyl Laboratories), which recognize distinct epitopes. Nuclear extracts from mouse testis were immunoprecipitated with protein A beads only (Mock) or protein A beads coupled to non-specific immunoglobulin G (IgG), anti-PDS5B #1, or anti-PDS5B #2. Testis nuclear extract proteins and the immunoprecipitates were probed with anti-PDS5B #1 (**a**) or anti-PDS5B #2 (**b**). The two antibodies provided similar results.

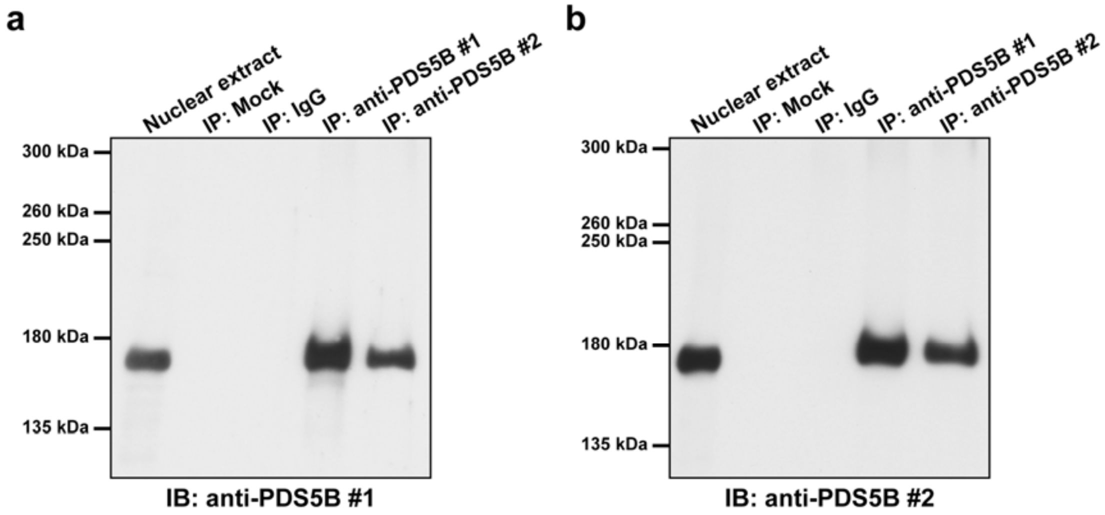

Supplementary Figure S2. PDS5B signals, but not SYCP3 signals, are inhibited by the peptide corresponding to the epitope of the anti-PDS5B antibody. Nuclear spreads of pachytene spermatocytes were stained with antibodies against SYCP3 (red) and PDS5B (green) in the presence of the indicated amounts of the peptide. Bar, 10 µm.

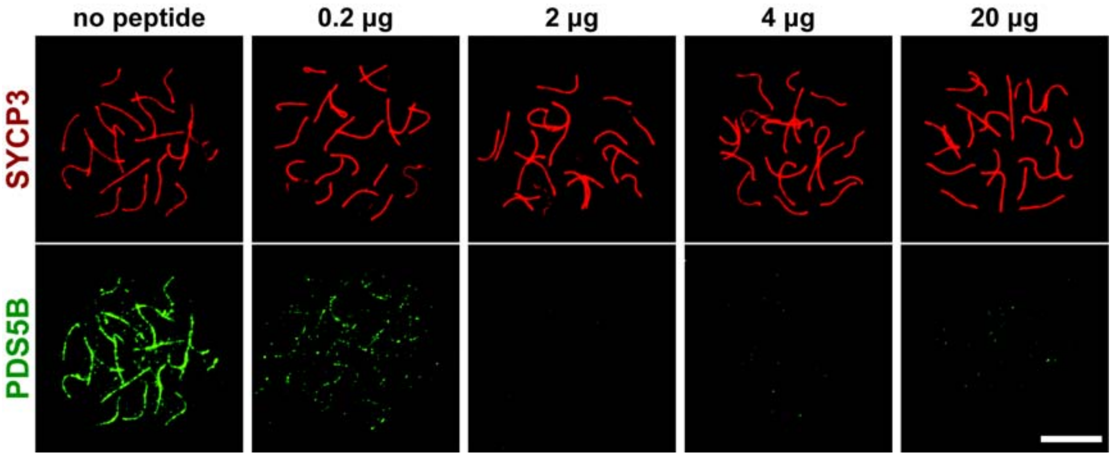

Supplement: Supplementary File 1 — PDF-Document (PDF, 132 KB) [file genes-01-00484-s001.pdf]
